# Factors associated with short- and long-term maintenance of sinus rhythm after successful elective electrical cardioversion

**DOI:** 10.3389/fcvm.2026.1951804

**Published:** 2026-10-13

**Authors:** Nikola N. Radovanović, Nemanja S. Branković, Nikola Vujadinović, Nebojša M. Mujović, Stefan Juričić, Olga Petrović, Srdjan Raspopović, Goran Milašinović

**Affiliations:** 1 Pacemaker Center, University Clinical Center of Serbia, Belgrade, Serbia; 2 Faculty of Medicine, University of Belgrade, Belgrade, Serbia; 3 Cardiology Clinic, University Clinical Center of Serbia, Belgrade, Serbia; 4 Department of Cardiology, University Clinical Hospital Center Zemun, Belgrade, Serbia

**Keywords:** associated factors, atrial fibrillation, atrial flutter, electrical cardioversion, sinus rhythm

## Abstract

**Introduction:**

Electrical cardioversion (ECV) effectively restores sinus rhythm in patients with persistent atrial fibrillation (AF) and atrial flutter (AFL), although arrhythmia recurrence is common. We aimed to identify factors associated with a higher probability of maintaining sinus rhythm one month after successful ECV, and after one year in those who were arrhythmia-free after the first month.

**Methods:**

This retrospective, single-center study included patients who underwent elective ECV for persistent AF or AFL between January 2011 and June 2023. Clinical, electrocardiographic, and echocardiographic characteristics were analyzed to identify parameters associated with the maintenance of sinus rhythm.

**Results:**

We included 297 ECV procedures. ECV was successful in 91.9% of patients and sinus rhythm was maintained in 89.7% of them at one month and in 40.3% at one year follow-up. Shorter arrhythmia duration (OR = 0.863, 95% CI: 0.76–0.98, *p* = 0.026) and ventricular pacing on the pre-procedural ECG (OR = 5.212, 95% CI: 1.16–23.48, *p* = 0.032) were associated with one-month success. Factors associated with one-year sinus rhythm maintenance in patients who were in sinus rhythm during monthly follow-up were smaller left atrial dimensions (OR = 0.817, 95%CI: 0.77–0.98, *p* = 0.022), the first-ever arrhythmia episode (OR = 3.320, 95%CI: 1.10–10.05, *p* = 0.034), lower CHA_2_DS_2_-VASc score (OR = 0.132, 95%CI: 0.03–0.54, *p* = 0.005), non-smoking status (OR = 0.120, 95%CI: 0.04–0.37, *p* < 0.001), no previous ECV (OR = 0.082, 95%CI: 0.01–0.47, *p* = 0.005), and antiarrhythmic therapy (OR = 2.486, 95%CI: 1.01–8.65, *p* = 0.05).

**Conclusions:**

ECV is a reasonable rhythm-control strategy in AF/AFL particularly when performed early after arrhythmia onset, in non-smokers and patients with fewer comorbidities, antiarrhythmic treatment, smaller left atrium and no prior ECV.

## Introduction

Electrical cardioversion (ECV) is a highly effective method for the acute restoration of sinus rhythm in patients with persistent atrial arrhythmias – atrial fibrillation (AF) and atrial flutter (AFL). Implementation of a rhythm control strategy often leads to significant improvement in AF/AFL-related symptoms (1, 2)*.* Beyond symptom control, maintaining sinus rhythm should also be pursued as it may contribute to reduced morbidity and mortality associated with AF/AFL (3). Despite the high success rate in terminating AF/AFL, long-term maintenance of sinus rhythm remains a significant clinical challenge. Therefore, identifying reliable predictors of sustained sinus rhythm following ECV is of considerable clinical value, as it may guide patient selection, determine treatment strategies, and improve long-term outcomes (4, 5).

Our goal was to determine the factors associated with a higher probability of maintaining sinus rhythm one month after successful elective ECV of persistent AF and AFL, and after one year in patients who were arrhythmia-free after the first month.

## Materials and methods

### Study design and patient selection

This is a retrospective, observational study, performed as a single-center experience among patients who underwent elective ECV for persistent AF or AFL at the Pacemaker Center, University Clinical Center of Serbia, between January 2011 and June 2023. Persistent atrial arrhythmia was defined as AF or AFL lasting for more than seven days. For patients who underwent multiple ECVs during this period, only the first procedure was included in the analysis. All patients received anticoagulation therapy, vitamin K antagonists (VKA) or direct oral anticoagulants (DOAC), for at least three weeks prior to ECV. Only patients with complete medical documentation were comprised, including discharge lists from hospitalization when the intervention was performed, and follow-up reports one month and one year after successful ECV.

### Definitions

We considered a patient to have ischemic heart disease if there was significant coronary artery disease (≥50% stenosis in 1 or more major epicardial coronary arteries), a history of myocardial infarction, previous percutaneous coronary intervention, or previous coronary bypass surgery. Valvular heart disease referred to stenosis or insufficiency of the valves with significant hemodynamic consequences, primarily in terms of structural remodeling of the atrium, as well as the existence of an prosthetic valve. Structural heart disease was the broadest term and included patients with coronary artery disease, advanced valvular disease, patients with hereditary or infiltrative cardiomyopathies and patients with congenital heart disease. Among the patients included in this study, current smokers were those who had smoked 100 cigarettes in their lifetime and reported smoking in the past month, while former smokers were those who had smoked 100 cigarettes in their lifetime, but denied smoking in the past month. Patients who did not meet the criteria for current or former smokers were marked as non-smokers. A positive family history of cardiovascular disease refers to the presence of coronary heart disease, cerebrovascular disease or peripheral arterial disease in first- or second-degree relatives. A diagnosis of heart failure in this study meant that the patient had symptoms of this disease, and in relation to left ventricular ejection fraction (LVEF), a division was made into heart failure with reduced LVEF (HFrEF, LVEF ≤ 40%) and with preserved LVEF (HFpEF, LVEF > 40%). Patients whose estimated glomerular filtration rate was less than 60 mL/min/1.73m^2^ were diagnosed with chronic kidney disease. The month when the presence of AF/AFL was first confirmed electrocardiographically was used to determine the total duration of atrial arrhythmia, which also corresponds to the duration of the current arrhythmia episode if sinus rhythm has not been recorded since then.

### Electrical cardioversion procedures (protocol)

This study was performed according to the ethical principles of the Helsinki Declaration. It was approved by the Ethics Committee of the University Clinical Center of Serbia (Approve Date: 07.05.2026., Ref Numb: 455/17) and each patient signed an informed consent form. All ECV procedures were performed in the operating room of the Pacemaker Center, University Clinical Center of Serbia, by an internal medicine specialist in the presence of an anesthesiologist, using a single protocol that did not change during the observed years. Pre-cardioversion assessments included a 12-lead ECG, non-invasive arterial pressure measurement, and routine blood tests. Transthoracic echocardiography (TTE) was performed within one month prior to hospital admission. Left atrial (LA) anteroposterior diameter was measured at end-systole in the parasternal long-axis view, perpendicular to the long axis of the aortic root, at the level of the aortic sinuses, using the leading-edge to leading-edge convention. Mitral and tricuspid regurgitation severity was obtained from the original TTE reports and was graded according to the routinely used semiquantitative scale as 0 (none), 1+ (mild), 2+ (mild-to-moderate), 3+ (moderate-to-severe), or 4+ (severe) (6, 7). On the day of the procedure, patients were instructed to fast for several hours beforehand. ECV was performed after intravenous administration of propofol, with continuous monitoring of blood pressure and oximetry during the procedure. Defibrillation electrodes were placed in the anterolateral position. The energy level of the initial shock was determined by the attending specialist based on the patient's body weight, arrhythmia type, and prior cardioversion history. If the first shock was ineffective, subsequent shocks were delivered at maximum energy, with up to three total shocks (minimum of two at full energy). In most patients with an implantable cardioverter-defibrillator (ICD), an initial internal maximum-energy shock was attempted. If unsuccessful, an external shock at maximum energy followed.

Restoration of sinus rhythm, if observed on the monitor, was immediately confirmed by a 12-lead ECG performed in the operating room. The 12-lead ECG was repeated upon the patient's return to the ward 30 min after the procedure. ECV was considered successful if sinus rhythm was confirmed on both ECG recordings. All patients were monitored for at least four hours following the procedure and were discharged the same day if no complications occurred.

### Patient follow-up

Scheduled follow-up visits were conducted one month and one year after successful ECV. All patients came to these examinations with the findings of 24 h ECG Holter monitoring, based on which therapy was adjusted as needed. At these controls, the CHA_2_DS_2_-VASc score was reassessed to make a decision regarding the continuation of anticoagulant therapy. In patients with cardiac implantable electronic device (CIED), device interrogation was also performed during scheduled visits, with a special emphasis on the analysis of newly recorded atrial high rate episodes in the memory of device with an atrial lead. If such episodes were recorded and lasted longer than 5 min, regardless of whether they were symptomatic, an active search for paroxysms of atrial arrhythmia was conducted by repeated 24–72 h ECG Holter monitoring. Emergency controls were performed in cases of symptoms, primarily palpitations. To confirm the recurrence of AF or AFL, a 12-lead ECG with a registered arrhythmia or an ECG Holter recording with a detected atrial arrhythmia lasting at least 30 s was required.

### Statistics

Statistical analyses were done using the software package SPSS version 17 (SPSS Inc., Chicago, IL, USA). Data are presented as mean (± sd), median (with range ​​from minimum to maximum values), or *n* (%) depending on data type. Normal distributions of data were tested by the Shapiro–Wilk test. When comparing categorical variables between groups, chi-square test and Fisher's exact test were used. Student's t-test was used for comparison of normally distributed continuous variables between examined groups, and when these variables did not follow a normal distribution the Mann–Whitney test was used. Factors associated with short- and long-term maintenance of sinus rhythm after successful ECV were examined by logistic regression analysis with the presentation of odds ratio (OR), 95% confidence interval of odds ratio (95% CI OR) and *p* values. Associated factors were sought among sociodemographic, clinical, electrocardiographic, echocardiographic, therapeutic, and ECV characteristics. First, a univariate analysis was performed with each parameter individually, and those that were statistically significant (entry treshold was *p* < 0.05) were included in the multivariate model after examining multicollinearity using Variance Inflation Factor (VIF) method, with treshold of 5 (variables with VIF over 5 were removed). When assessing factors associated with maintenance of sinus rhythm in the first month after successful ECV, all significant variables from the univariable were also in the multivariable model, and for the endpoint associated with long-term maintenance of sinus rhythm in patients who were in sinus rhythm during the monthly follow-up, there were some changes. More precisely, instead of including the variables “antiarrhythmic drugs before ECV”, “antiarrhythmic drugs at discharge”, “amiodarone before ECV”, “amiodarone at discharge”, and “amiodarone both before ECV and at discharge”, only the variable “antiarrhythmic therapy before ECV and at discharge” was included in the multivariable model. For smoking, a new categorical variable was created, categorizing participants as smokers (current or former smokers) and non-smokers. “Total duration of atrial arrhythmia” was excluded from the multivariable model because VIF exceeded 5. Both endpoint analysis were adjusted for age, gender, previous ECV, and number of comorbidities. In order not to overfit the model we choose Backward Wald method with removal threshold of 0.10. The last step with Cox & Snell R2 measure of model fit was reported. Event-free survival was estimated using Kaplan–Meier analysis and compared between groups using the log-rank test. For all tests, probability values less than or equal to 0.05 were considered as statistically significant.

## Results

### Study population

Over a period of 13 years and 6 months, a total of 348 elective ECV procedures for persistent AF or AFL were performed at the Pacemaker Center of the University Clinical Center of Serbia. This study included 297 consecutive eligible ECV procedures, each performed on a different patient. Thirty-three procedures were excluded due to incomplete medical documentation, 14 due to the lack of a discharge list from hospitalization when the intervention was performed, and 19 due to the unavailability of follow-up reports one month and/or one year after ECV. Eighteen procedures were repeated ECVs in patients who had already undergone the first ECV within the defined study period, so they were not included in the analysis (Figure 1). The mean age of the included patients was 63.9 ± 11.4 years, with a predominance of males (75.4%).

**Figure 1:**
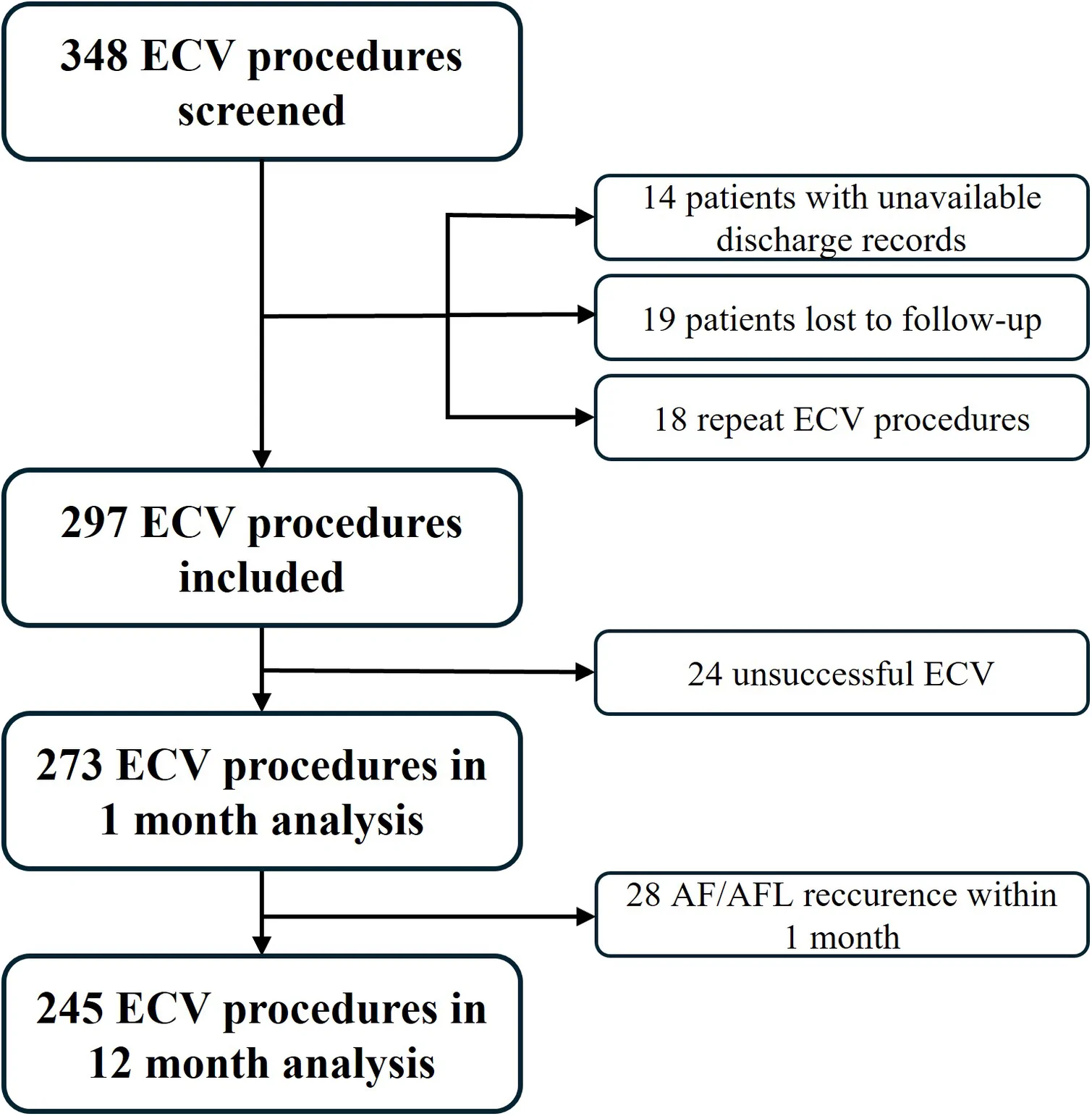
Flow chart.

### Clinical characteristics of patients and procedure success

ECV was successful in 273 patients (91.9%). In two-thirds of patients, the reason for elective ECV was AF, in the remaining AFL. The procedure was statistically significantly more successful in patients with AFL (*p* = 0.03). The median time from first arrhythmia diagnosis to attempted ECV was 14 months, with the shortest period lasting one month and the longest 266 months. The median duration of the current episode of atrial arrhythmia was 3 months, with a range from 1 to 24 months. The current episode of arrhythmia was also the first episode in almost half of the patients (42.4%). In the overall study population, LVEF was 53.6 ± 11.4%, the average LA dimension 45.8 ± 6.0 mm, and approximately two-thirds of patients exhibited mild mitral and/or tricuspid regurgitation. The prevalence of comorbidities and cardiovascular risk factors in the studied population is presented in Table 1. The pharmacological therapy prescribed at hospital discharge following successful ECV did not differ significantly from the pre-procedural regimen. As expected, all patients were receiving oral anticoagulant therapy, more commonly VKA (67.7%). Beta-blockers were prescribed to nearly all patients, and the majority were also receiving an antiarrhythmic agent, as well as renin-angiotensin system (RAS) inhibitor. More than half of the patients were being treated with diuretics, primarily thiazide-type diuretics, and statin therapy was used in approximately 40% of the study population. Nearly half of the patients included in this study had a CIED implanted. The most common device was an anti-bradycardia pacemaker, implanted in 108 patients. This was followed by 28 ICDs, including patients with cardiac resynchronization therapy defibrillators (CRT-D). Additionally, 18 patients had cardiac resynchronization therapy (CRT) pacemakers, with or without defibrillator function. The fact that the patient had an implanted device before ECV did not significantly affect the success of this procedure, nor did the type of device or the time that elapsed from device implantation to the intervention (Table 2). Nearly a quarter of patients had at least one ECV preceding the studied procedure. In 85 patients (28.6%), ventricular pacing was registered on the ECG before the procedure, and this parameter did not differ significantly between patients with successful ECV and those who remained in atrial arrhythmia after the procedure (*p* = 0.140). The characteristics of the shock that led to electrical cardioversion are shown in Table 3. Initial maximum-energy internal shock was attempted in 20 patients with implanted ICDs and was successful in 15 cases. In the remaining 5 patients, an external shock at maximum energy followed, up to a maximum of 3 shocks in a row, with sinus rhythm achieved in 3 more patients.

**Table 1:** Indications for the procedure and prevalence of comorbidities and cardiovascular risk factors.

Comorbidity		Number of patients (%)
Indication for procedure	Atrial fibrillation	201 (67.7)
	Atrial flutter	96 (32.3)
Hypertension		255 (85.9)
Diabetes mellitus		51 (17.2)
Dyslipidemia		99 (33.3)
Chronic obstructive pulmonary disease		28 (9.4)
Smoking status	Smoker (current + former)	161 (54.2)
	Current smoker	40 (24.8)
	Former smoker	121 (75.2)
Family history of cardiovascular disease		126 (42.4)
Ischemic heart disease		57 (19.2)
Heart failure	Yes (HFpEF + HFrEF)	66 (22.2)
	HFpEF	12 (4.0)
	HFrEF	54 (18.2)
Previous TIA and/or CVI		26 (8.8)
Chronic kidney disease		56 (18.9)
Thyroid dysfunction	Yes (hypothyroidism + hyperthyroidism)	41 (13.8)
	Hypothyroidism	32 (10.8)
	Hyperthyroidism	9 (3.0)
Structural heart disease		119 (40.1)
Dilated cardiomyopathy		54 (18.2)
Hypertrophic cardiomyopathy		6 (2.0)
Valvular heart disease		21 (7.1)
Congenital heart disease		5 (1.7)

**Table 2:** Success of elective electrical cardioversion in patients who had an implantable electronic cardiac device implanted prior to the procedure.

Parameter	All patients (%) (*N* = 297)	Successful ECV		*p* value
		Yes (*N* = 273)	No (*N* = 24)	
Implanted CIED	144 (48.5)	129 (47.3)	15 (62.5)	0.152
Implanted anti-bradycardia PM	108 (36.4)	96 (35.2)	12 (50.0)	0.147
Implanted ICD/CRT-D	28 (9.4)	26 (9.5)	2 (8.3)	0.848
Implanted CRT (P/D)	18 (6.1)	16 (5.9)	2 (8.3)	0.626
Time from device implantation to ECV (months), med (min-max)	11 (1–354)	11 (1–354)	8 (1–241)	0.416

CIED, cardiac implantable electronic device; ECV, electrical cardioversion.

**Table 3:** Characteristics of the shock that led to electrical cardioversion.

Parameter	Value	Successful ECV *n* (%) (*N* = 273)
Shock energy that led to cardioversion	maximum	183 (66.7)
	less than maximum	90 (33.3)
Shock energy that led to cardioversion	100J	1 (0.4)
	150J	25 (9.2)
	200J	64 (23.5)
	270J	165 (60.3)
	360J	3 (1.1)
	internal maximum shock	15 (5.5)
Number of failed shocks preceding a successful one	0	227 (83.2)
	1	37 (13.6)
	2	9 (3.3)

ECV, electrical cardioversion.

### Factors associated with a higher probability of maintaining sinus rhythm in the first month after successful electrical cardioversion

Among the 273 patients in whom ECV was initially successful, 245 patients (89.7%) maintained sinus rhythm during the first month of follow-up (in this statistical model the number of observations was 273, number of outcome events was 28, and the missing data was 25).

Short-term maintenance of sinus rhythm was statistically more common in patients with smaller LA dimensions, larger LVEF, shorter total duration of arrhythmia, and current episode. Patients who had structural heart disease (SCD), who had alredy undergone ECV, and who used acetylsalicylic acid (ASA) before or after the intervention had significantly more arrhythmia recurrences in the first month after a successful procedure. All patients who used flecainide before the successful procedure, were still in sinus rhythm after one month. The results are shown in Tables 4, 5.

**Table 4:** Comorbidities and pre-procedural echocardiographic findings.

Parameter	All patients (%) (*N* = 273)	Maintenance of sinus rhythm at one month after successful ECV		*p* value	All patients (%) (*N* = 245)	Maintenance of sinus rhythm one year after successful ECV in patients who were in sinus rhythm at one month		*p* value
		Yes (%) (*N* = 245)	No (%) (*N* = 28)			Yes (%) (*N* = 110)	No (%) (*N* = 135)	
AF as indication for ECV	180 (65.9)	160 (65.3)	20 (71.4)	0.517	160 (65.3)	71 (64.5)	89 (65.9)	0.821
Hypertension	233 (85.3)	208 (84.9)	25 (89.3)	0.534	208 (84.9)	90 (81.8)	118 (87.4)	0.224
Diabetes melitus	47 (17.2)	43 (17.6)	4 (14.3)	0.665	43 (17.6)	19 (17.3)	24 (17.8)	0.918
Hyperlipidemia	92 (33.7)	83 (33.9)	9 (32.1)	0.854	83 (33.9)	26 (23.6)	57 (42.2)	0.002
COPD	24 (8.8)	23 (9.4)	1 (3.6)	0.303	23 (9.4)	8 (7.3)	15 (11.1)	0.306
Family history of CVD	112 (41.0)	98 (40.0)	14 (50.0)	0.308	98 (40.0)	29 (26.4)	69 (51.1)	<0.001
Coronary artery disease	54 (19.8)	45 (18.4)	9 (32.1)	0.083	45 (18.4)	18 (16.4)	27 (20.0)	0.465
Smoking (current + former)	144 (52.7)	128 (52.2)	16 (57.1)	0.623	128 (52.2)	38 (34.5)	90 (66.7)	<0.001
Heart failure	55 (20.1)	48 (19.6)	7 (25.0)	0.499	48 (19.6)	16 (14.5)	32 (23.7)	0.072
Chronic kidney disease	53 (19.4)	45 (18.4)	8 (28.6)	0.196	45 (18.4)	6 (5.5)	39 (28.9)	<0.001
CHA_2_DS₂-VASc ≤ 3	211 (77.3)	190 (77.6)	21 (75.0)	0.760	55 (22.5)	16 (14.5)	39 (28.9)	0.007
Thyroid dysfunction	40 (14.7)	33 (13.5)	7 (25.0)	0.102	33 (13.5)	7 (6.4)	26 (19.3)	0.003
Structural heart disease	105 (38.5)	89 (36.3)	16 (57.1)	0.032	89 (36.3)	32 (29.1)	57 (42.2)	0.034
Dilated cardiomyopathy	46 (16.8)	38 (15.5)	8 (28.6)	0.080	38 (15.5)	13 (11.8)	25 (18.5)	0.150
Hypertrophic cardiomyopathy	6 (2.2)	4 (1.6)	2 (7.1)	0.060	4 (1.6)	0 (0.0)	4 (3.0)	0.069
Valvular heart disease	17 (6.2)	14 (5.7)	3 (10.7)	0.300	14 (5.7)	4 (3.6)	10 (7.4)	0.206
Congenital heart disease	4 (1.5)	4 (1.6)	0 (0.0)	0.496	4 (1.6)	3 (2.7)	1 (0.7)	0.222
LVEF (%), mean ± SD	54.0 ± 11.2	54.2 ± 10.9	51.9 ± 13.4	0.036	54.2 ± 10.9	55.2 ± 11.1	53.4 ± 10.7	0.485
LA diameter (mm), mean ± SD	45.7 ± 6.0	45.4 ± 5.8	48.1 ± 6.9	0.025	45.4 ± 5.8	44.0 ± 5.0	46.6 ± 6.2	<0.001
Total duration of AA (months), mean ± SD	30.7 ± 42.1	29.2 ± 42.5	44.3 ± 36.2	<0.001	29.2 ± 42.5	17.0 ± 34.2	39.2 ± 46.1	0.111
Current AA episode duration (months), mean ± SD	4.7 ± 4.2	4.5 ± 4.1	6.2 ± 5.1	0.002	4.5 ± 4.1	3.9 ± 3.0	5.0 ± 4.7	0.457
Previous ECV	66 (24.3)	55 (22.5)	11 (39.3)	0.050	55 (22.5)	13 (11.9)	42 (31.1)	<0.001
Implanted CIED	129 (47.3)	118 (48.2)	11 (39.3)	0.373	118 (48.2)	46 (41.8)	72 (53.3)	0.073
Ventricular pacing on the preprocedural ECG	75 (27.5)	70 (28.6)	5 (17.9)	0.229	70 (28.6)	29 (26.4)	41 (30.4)	0.490

AF, atrial fibrillation; COPD, chronic obstrucitve pulmonary disease; CVD, cardiovascular disease; LVEF, left ventricular ejection fraction; LA, left atrium; AA, atrial arrhythmia; ECV, electrical cardioversion; CIED, cardiac implantable electronic device.

**Table 5:** Medication use before ECV and at hospital discharge.

Parameter	All patients (%) (*N* = 273)	Maintenance of sinus rhythm at one month after successful ECV		*p* value	All patients (%) (*N* = 245)	Maintenance of sinus rhythm one year after successful ECV in patients who were in sinus rhythm at one month		*p* value
		Yes (%) (*N* = 245)	No (%) (*N* = 28)			Yes (%) (*N* = 110)	No (%) (*N* = 135)	
Before ECV								
Beta-blockers	264 (96.7)	238 (97.1)	26 (92.9)	0.229	238 (97.1)	107 (97.3)	131 (97.0)	0.912
RAS inhibitors	212 (77.7)	191 (78.0)	21 (75.0)	0.722	191 (78.0)	79 (71.8)	112 (83.0)	0.036
Furosemid	61 (22.3)	53 (21.6)	8 (28.6)	0.404	53 (21.6)	23 (20.9)	30 (22.2)	0.804
MRA	50 (18.3)	43 (17.6)	7 (25.0)	0.334	43 (17.6)	18 (16.4)	25 (18.5)	0.659
Antiarrhythmic drugs	239 (87.5)	215 (87.8)	24 (85.7)	0.757	215 (87.8)	103 (93.6)	112 (83.0)	0.011
Amiodarone	179 (65.6)	160 (65.3)	19 (67.9)	0.788	160 (65.3)	80 (72.7)	80 (59.3)	0.028
Propafenone	49 (17.9)	44 (18.0)	5 (17.9)	0.989	44 (18.0)	18 (16.4)	26 (19.3)	0.557
Flecainide	10 (3.7)	10 (4.1)	0 (0.0)	0.276	10 (4.1)	5 (4.5)	5 (3.7)	0.740
Acetylsalicylic acid	21 (7.7)	16 (6.5)	5 (17.9)	0.033	16 (6.5)	6 (5.5)	10 (7.4)	0.538
Statins	105 (38.5)	90 (36.7)	15 (53.6)	0.083	90 (36.7)	30 (27.3)	60 (44.4)	0.006
At hospital discharge after ECV								
Beta-blockers	266 (97.4)	238 (97.1)	28 (100.0)	0.365	238 (97.1)	108 (98.2)	130 (96.3)	0.378
RAS inhibitors	209 (76.6)	188 (76.7)	21 (75.0)	0.837	188 (76.7)	78 (70.9)	110 (81.5)	0.051
Furosemid	62 (22.7)	53 (21.6)	9 (32.1)	0.209	53 (21.6)	22 (20.0)	31 (23.0)	0.575
MRA	48 (17.6)	41 (16.7)	7 (25.0)	0.276	41 (16.7)	16 (14.5)	25 (18.5)	0.407
Antiarrhythmic drugs	250 (91.6)	225 (91.8)	25 (89.3)	0.645	225 (91.8)	106 (96.4)	119 (88.1)	0.019
Amiodarone	186 (68.1)	167 (68.2)	19 (67.9)	0.974	167 (68.2)	82 (74.5)	85 (63.0)	0.053
Propafenone	53 (19.4)	47 (19.2)	6 (21.4)	0.776	47 (19.2)	19 (17.3)	28 (20.7)	0.493
Flecainide	10 (3.7)	10 (4.1)	0 (0.0)	0.276	10 (4.1)	5 (4.5)	5 (3.7)	0.740
Acetylsalicylic acid	20 (7.3)	15 (6.1)	5 (17.9)	0.024	15 (6.1)	6 (5.5)	9 (6.7)	0.694
Statins	105 (38.5)	91 (37.1)	14 (50.0)	0.185	91 (37.1)	30 (27.3)	61 (45.2)	0.004

ECV, electrical cardioversion; RAS, renin-angiotensin system; MRA, mineralocorticoid receptor antagonists.

Several factors were identified as probably associated with a higher probability of maintaining sinus rhythm at one month: shorter duration of the atrial arrhythmia episode leading to ECV, presence of ventricular pacing on the pre-procedural 12-lead ECG, smaller LA dimensions, and absence of ASA use before and after the procedure (Table 6).

**Table 6:** Factors associated with a higher probability of maintaining sinus rhythm in the first month after successful electrical cardioversion.

Factor	Univariate logistic regression analysis			Multivariate logistic regression analysis (Backward Wald method, last step, Cox & Snell R2 = 23.8%)			
	OR	95%CI OR	*p*	OR	95%CI OR	*p*	*p* *
Duration of the atrial arrhythmia episode leading to ECV	0.922	0.86–0.99	0.035	0.863	0.76–0.98	0.026	0.011
Ventricular pacing on pre-procedural ECG	4.118	1.07–15.85	0.040	5.212	1.16–23.48	0.032	0.029
Left atrial size	0.934	0.88–0.99	0.028	0.915	0.83–1.00	0.060	0.168
ASA therapy before ECV	0.307	0.10–0.92	0.035				
ASA therapy at discharge after successful ECV	0.287	0.10–0.86	0.026				

ECV, electrical cardioversion; ASA, acetylsalicylic acid.

* adjusted for age, gender, previous ECV, number of comorbidities.

In multivariate analysis, two factors were found to be independently associated with sinus rhythm maintenance at one month: shorter duration of the current episode of atrial arrhythmia (OR = 0.863, 95% CI: 0.76–0.98, *p* = 0.026), and ventricular pacing observed on the pre-procedural ECG (OR = 5.212, 95% CI: 1.16–23.48, *p* = 0.032) (Table 6).

### Factors associated with a higher probability of maintaining sinus rhythm one year after successful electrical cardioversion in patients who were arrhythmia-free in the first month after the procedure

At one-year follow-up, sinus rhythm was maintained in 110 patients, representing 40.3% of all patients with initially successful electrical cardioversion, and 44.9% of those who were in sinus rhythm at one-month follow-up (in this statistical model the number of observations was 245, number of outcome events was 135, and the missing data was 52).

As with short-term, long-term maintenance of sinus rhythm was significantly less common in patients with SCD, who had previously undergone ECV, and had larger LA dimensions. Also, analysis of one-year follow-up showed that arrhythmia recurrence occurs more often if the patient has various risk factors and associated diseases such as smoking, family burden for cardiovascular diseases, dyslipidemia, chronic kidney disease, thyroid dysfunction. The results indicate the importance of medications administered before and after elective ECV for maintaining sinus rhythm. Thus, patients who used statins or antiarrhythmics before the procedure and at discharge, and separately amiodarone, as well as RAS inhibitors before the procedure, were significantly more likely to maintain sinus rhythm in the long term. No patient with hypertrophic cardiomyopathy had sinus rhythm after one year of follow-up (Tables 4, 5).

At 12 months, the arrhythmia recurrence rate was not significantly different in patients with AF compared with those with AFL as an indication for ECV, analyzed in patients who had confirmed short-term maintenance of sinus rhythm (Figure 2).

**Figure 2:**
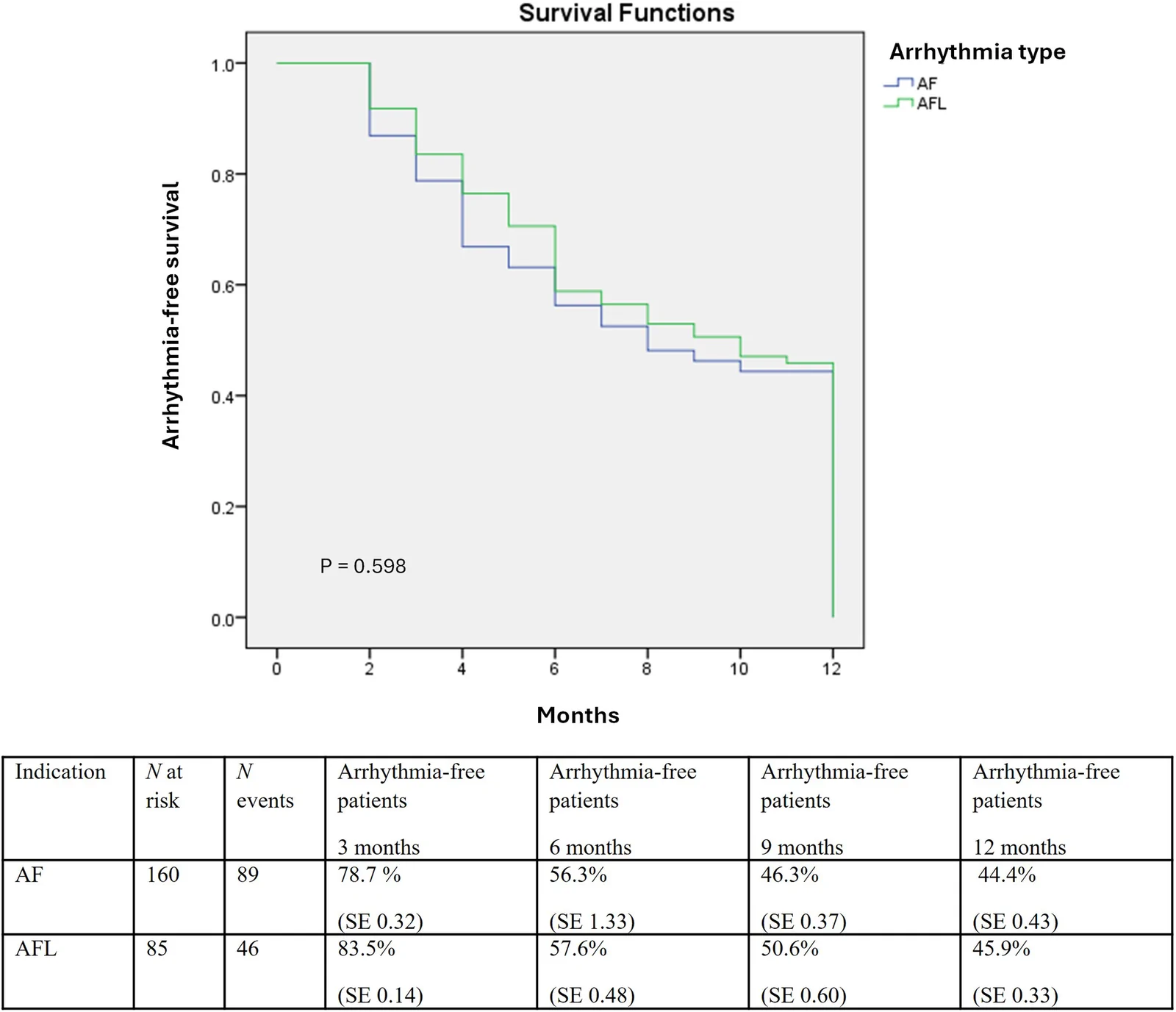
Kaplan–Meier estimates of time to arrhythmia recurrence according to atrial arrhythmia type (analyzed in patients who had confirmed short-term maintenance of sinus rhythm).

Several clinical and echocardiographic parameters have been identified as probably associated with the long-term success of ECV (Table 7). Among the factors related to arrhythmia, shorter total duration of atrial arrhythmia, as well as the current episode, and parameters indicating that it was the first-ever arrhythmic episode, i.e., that there were no previous ECVs, were highlighted. Within comorbidities and risk factors, univariate regression analysis identified dyslipidemia, heredity, thyroid dysfunction, chronic kidney disease, SCD, non-smoking status, and a CHA_2_DS_2_-VASc score less than or equal to 3 as parameters likely associated with long-term maintenance of sinus rhythm. Furthermore, the use of antiarrhythmics both before the procedure and at discharge, as well as the absence of RAS inhibitors and statins in pre-ECV therapy, were likely associated with the long-term success of elective ECV. Echocardiographic parameters such as smaller LA dimensions and mild mitral and tricuspid regurgitation were also found in the same group of factors. In patients with CIED, the presence of a CRT or ICD, as well as the use of a strategy involving atrial lead implantation during CIED insertion in patients with persistent atrial fibrillation with subsequent elective ECV, were likely associated with the occurrence of arrhythmia recurrence during one-year follow-up.

**Table 7:** Factors associated with a higher probability of maintaining sinus rhythm after one year of electrical cardioversion in patients who were in sinus rhythm during monthly follow-up.

Factor		Univariate logistic regression analysis			Multivariate logistic regression analysis (Backward Wald method, Last step, Cox & Snell R2 = 40.1%)			
		OR	95%CI OR	*p*	OR	95%CI OR	*p*	*p* *
Total duration of atrial arrhythmia		0.980	0.97–0.99	<0.001				
Duration of the atrial arrhythmia episode leading to ECV		0.929	0.87–0.99	0.038	0.752	0.64–1.01	0.051	0.054
First episode of arrhythmia		3.102	1.84–5.24	<0.001	3.320	1.10–10.05	0.034	0.033
Left atrial size		0.919	0.88–0.97	0.001	0.817	0.77–0.98	0.022	0.023
Mitral regurgitation	Mild	Ref.						
	Mild-to-moderate	0.299	0.17–0.54	<0.001				
	Moderate-to-severe	0.236	0.06–0.89	0.034				
Tricuspid regurgitation	Mild	Ref.						
	Mild-to-moderate	0.322	0.17–0.60	<0.001				
	Moderate-to-severe	0.251	0.05–1.24	0.090				
Dyslipidemia		0.424	0.24–0.74	0.002				
Smoking status (current and former)		0.264	0.16–0.45	<0.001	0.120	0.04–0.37	<0.001	<0.001
Former smoker		0.242	0.14–0.43	<0.001				
Family history of cardiovascular disease		0.342	0.20–0.59	<0.001				
Thyroid dysfuction		0.285	0.12–0.69	0.005				
Hypothyroidism		0.205	0.07–0.62	0.005				
Chronic kidney disease		0.142	0.06–0.35	<0.001				
CHA_2_DS_2_-VASc score >3		0.419	0.22–0.80	0.008	0.132	0.03–0.54	0.005	0.004
Previous ECV		0.300	0.15–0.59	0.001	0.082	0.01–0.47	0.005	0.009
Number of prior ECVs	None	Ref.						
	1	0.364	0.15–0.86	0.021				
	2	0.429	0.11–0.71	0.229				
	>2	0.125	0.02–1.02	0.052				
Structural heart disease		0.561	0.33–0.96	0.034				
RAS inhibitors before ECV		0.523	0.28–0.97	0.038				
Antiarrhythmic therapy before ECV		3.022	1.24–7.34	0.015				
Amiodarone before ECV		1.833	1.07–3.15	0.028				
Statin therapy before ECV		0.469	0.27–0.80	0.006				
Implanted ICD before ECV		0.310	0.11–0.86	0.025				
Implanted CRT before ECV		0.094	0.01–0.74	0.024				
CIED implanted during arrhythmia followed by ECV		2.529	1.18–5.42	0.017				
Amiodarone before ECV and at discharge		2.039	1.19–3.49	0.009				
Antiarrhythmic therapy at discharge after ECV		3.563	1.16–10.99	0.027				
Antiarrhythmic therapy before ECV and at discharge		2.898	1.25–6.72	0.013	2.486	1.01–8.65	0.050	0.052
Statin therapy after ECV		0.455	0.27–0.78	0.004				

ECV, electrical cardioversion; RAS, renin-angiotensin system; CIED, cardiac implantable electronic device.

* adjusted for age, gender, previous ECV, number of comorbidities.

Of these factors, the following were identified as independently associated with maintenance of sinus rhythm one year after successful electrical cardioversion in patients who were arrhythmia-free in the first month after the procedure: smaller LA dimensions (OR = 0.817, 95%CI: 0.77–0.98, *p* = 0.022), the first-ever arrhythmia episode (OR = 3.320, 95%CI: 1.10–10.05, *p* = 0.034), non-smoking status (OR = 0.120, 95%CI: 0.04–0.37, *p* < 0.001), CHA_2_DS_2_-VASc score less than or equal to 3 (OR = 0.132, 95%CI: 0.03–0.54, *p* = 0.005), absence of previous ECV (OR = 0.082, 95%CI: 0.01–0.47, *p* = 0.005) and the use of antiarrhythmics both before successful ECV and at discharge (OR = 2.486, 95%CI: 1.01–8.65, *p* = 0.05) (Table 7).

## Discussion

Although the importance of elective ECV has changed in recent years, this procedure remains part of the rhythm control strategy in patients with atrial arrhythmias. Moreover, ECV has even gained a new role in the selection of patients for catheter ablation (3, 8). This procedure is used to assess the symptom-rhythm correlation in patients who deny significant symptoms or in whom we are unsure whether the arrhythmia is a crucial contributor to the symptoms development. If their quality of life improves significantly after successful elective ECV, they become good candidates for catheter ablation (3, 9). Also, ECV may be important in the evaluation of potentially reversible ventricular dysfunction associated with atrial fibrillation (3).

This study attempts to answer a key question: what should be the profile of a patient who has the greatest chance of long-term benefit from elective ECV? To reach a valid conclusion, a considerable number of procedures were comprised, and by including only the first procedure in patients who underwent multiple ECVs during a defined period, bias in the analysis is avoided.

### Maintenance of sinus rhythm one month after successful electrical cardioversion

Short-term maintenance of sinus rhythm after successful elective ECV was achieved in almost 90% of patients included in this research, which is a notably more favorable outcome compared to previously conducted, similarly designed studies (10, 11). The degree of atrial remodeling, which is directly related to increased electrical instability, is decisive for early re-development of arrhythmia (12). Therefore, the duration of the atrial arrhythmia episode leading to ECV is crucial, as well as its total duration and atrial size, parameters that were identified in this study as possible and independent associated factors of short-term maintenance of sinus rhythm. In this context, all chronic conditions and diseases that lead to left atrial remodeling, such as mitral valve disease or left ventricular dysfunction, may also be significant (13, 14). In our study, maintenance of sinus rhythm one month after successful ECV was statistically significantly more common in patients with higher LVEF, and less often in those with SCD. Most patients in this study underwent ECV during a time when guidelines differed from current practice, which explains the frequent use of antiplatelets alongside anticoagulants, especially in those with coronary artery disease and atrial arrhythmia. Although the literature reports inconsistent findings regarding the impact of coronary artery disease on post-cardioversion rhythm outcomes, a large study involving nearly 3.000 patients and approximately 7.000 cardioversion procedures demonstrated that the one-month sinus rhythm maintenance rate was significantly lower in patients with known vascular disease, primarily coronary (15). Therefore, our finding that ASA use was associated with early arrhythmia recurrence should be interpreted as a surrogate marker for underlying vascular disease, a known predictor of unsuccessful sinus rhythm maintenance after cardioversion. An interesting finding in this research is the association between ventricular pacing and short-term maintenance of sinus rhythm after ECV. Notably, this connection was not observed across all patients with implanted CIED, but was specifically limited to those whose indication for cardioversion was atrial arrhythmia with slow ventricular conduction, which required ventricular pacing. This observation aligns with the study by Hellman et al., which found that a ventricular rate above 60 bpm during AF was an independent predictor of arrhythmia recurrence within one month post-cardioversion (10). The lower ventricular rate reflects reduced sympathetic activity, the absence of overt cardiac decompensation, and possibly a predominance of atrial pacing after restoration of sinus rhythm, which, at least in the short term, could contribute to greater rhythm stability. However, this association between ventricular pacing and the absence of arrhythmia recurrence during the first month post-procedure should be considered exploratory, due to the limited number of events and the resulting model instability. An important finding is that every patient who used flecainide before successful ECV or at discharge was still in sinus rhythm one month after the procedure. With longer follow-up, the significance of flecainide usage is lost, raising the open question of how long antiarrhythmics should be prescribed after successful ECV. There is currently evidence that flecainide is more effective in preventing recurrence of atrial arrhythmias when taken continuously after cardioversion for at least six months, compared with a strategy of stopping the drug one month after the procedure (16, 17).

### Maintenance of sinus rhythm one year after successful electrical cardioversion in patients who were arrhythmia-free in the first month after the procedure

In our study, approximately 40% of patients maintained sinus rhythm one year after successful ECV for persistent AF or AFL. Among those who were in sinus rhythm at the one-month follow-up, indicating no early arrhythmia recurrence, the one-year maintenance rate was about 45%. Compared with the outcomes of large registries, this result is worse, but compared with the outcomes of studies comparable in design, it is quite favorable (11, 18, 19).

Atrial fibrillation is a progressive disease, characterized by electrical and structural remodeling of the LA, which over time increases the atrial substrate's vulnerability to arrhythmia recurrence, even after successful restoration of sinus rhythm (18, 20). The progression of AF is influenced by both the duration of the episode leading to ECV and the total arrhythmia burden, which includes not only the known duration of arrhythmia, but also its frequency and cumulative impact over time (19, 20). The results of this research indicated that shorter duration of the arrhythmia episode that is the reason for ECV, especially when it is the first episode of arrhythmia at all, as well as smaller LA dimensions, are independetly associated with long-term maintenance of sinus rhythm. It is difficult to set strict cutoff values, but previous studies have shown that the duration of an AF episode longer than 6 months is associated with a statistically significantly higher rate of arrhythmia recurrence during long-term follow-up (21). The same applies to the LA dimensions. In most studies, LA diameter of over 45 millimeters has been defined as the treshold value above which this parameter becomes independent predictor of atrial arrhythmia recurrence during the first year of successful cardioversion (22). However, current practice is moving toward the use of left atrial volume indexed to body surface area (LAVI), which offers a more accurate and standardized assessment of atrial size and its pathological significance. An increase in volume of only 1 mL/m^2^ increases the risk of arrhythmia recurrence by 6% (23).

In this study, patients who had previously undergone ECV not only had a statistically significantly higher rate of arrhythmia recurrence during the one-year follow-up, but this parameter was also recognized as an independent negative associated factor of long-term sinus rhythm maintenance. Although some authors advocate an aggressive approach in the form of repeated ECVs, especially in the case of early recurrence of arrhythmia, studies have showed that if the arrhythmia recurs after the second ECV, it is reasonable not to insist on further attempts because the third procedure results in sinus rhythm that is maintained for a long time in less than 15% of patients (4). A history of previous ECV has also been shown to significantly reduce the likelihood of spontaneous conversion to sinus rhythm, which occurs in over 40% of patients within the first 6 h of emergency department admission for symptomatic AF, and for whose prediction we currently have a probability score of reasonable accuracy (24, 25). It is well established that various comorbidities significantly reduce the likelihood of long-term maintenance of sinus rhythm. In this research, as in some others, smoking, family burden for cardiovascular diseases, dyslipidemia, chronic kidney disease, thyroid dysfunction, SHD were significantly more common in patients with recurrent arrhythmias, and these risk factors and associated diseases were also identified as possible, or independent, negative associated factors of long-term maintenance of sinus rhythm (19, 20, 26–28). Also, in our study, the CHA_2_DS_2_-VASc score was identified as an independent associated factor of long-term sinus rhythm maintenance. It is important to note that the stated threshold value of 3 for this score was not prespecified but was established subsequently based on the results of the data analysis. This finding further underscores the prognostic value of overall cardiovascular risk burden, and complements the results of previous studies that have shown that a higher CHA_2_DS_2_-VASc score is directly associated with new-onset of AF, with unsuccessful electrical cardioversion of persistent AF, and predicts the risk of early AF recurrence in the first 30 days after electrical or pharmacological cardioversion (29–31).

For the majority of antiarrhythmic drugs, there is a clear evidence supporting their role in significantly reducing the recurrence of atrial arrhythmia following successful ECV. A large meta-analysis demonstrated that the pre-procedural initiation of class Ic antiarrhythmics was associated with a statistically significant prolongation of sinus rhythm maintenance post-cardioversion. This effect was observed both at the class level and when flecainide and propafenone were analyzed individually (16). Amiodarone, when introduced prior to elective ECV, has been shown to enhance the success rate of the procedure to a degree comparable to class Ic antiarrhythmic agents. Moreover, evidence from meta-analyses suggests that amiodarone may be more effective than class Ic drugs in achieving long-term maintenance of sinus rhythm (32, 33). However, studies have showed that during 12 months of amiodarone use, 15%–20% of patients develop one of the drug-related complications (34, 35). Therefore, if we exclude patients in whom it is possible to prescribe exclusively amiodarone due to concomitant diseases, in others, potential benefits and side effects should always be carefully weighed when deciding which antiarrhythmic to administer periprocedurally. In this study, the use of antiarrhythmics before elective ECV and at discharge was on the verge of statistical significance to be independetly associated with preservation of sinus rhythm one year after the procedure, and patients who used amiodarone periprocedurally were more likely to be free of arrhythmia recurrence in the long term.

A frequent clinical dilemma for device implanters is whether to implant an atrial lead in a patient who has persistent AF at the time of permanent pacemaker implantation, with the intention of pursuing sinus rhythm restoration post-implantation, or to implant only a ventricular lead and accept the atrial arrhythmia as permanent (36). The findings of our study suggest that performing elective ECV in patients who had a permanent pacemaker system including an atrial lead implanted during ongoing persistent AF is associated with poorer long-term outcomes.

Furthermore, our data indicate that the presence of ICD or CRT devices, as well as the use of RAS inhibitors and statins, are associated with a higher incidence of arrhythmia recurrence during one-year follow-up. In addition, no patient with hypertrophic cardiomyopathy after successful elective ECV has maintained sinus rhythm in the long term. These findings underscore the impact of underlying structural heart disease on the long-term success of rhythm control strategies. In such patients, even after successful ECV, there is a high likelihood of arrhythmia recurrence, and they should be promptly referred for evaluation for catheter ablation as a potential long-term solution.

### Study limitations

Selection bias was not completely avoided in this study. We excluded patients with incomplete follow-up data, and we know that patients with recurrent arrhythmia or poor clinical status are more likely to be lost to follow-up in clinical practice. The long recruitment period is another limitation of this study. During the inclusion period of more than 13 years, there have been significant changes in medical and invasive therapy of AF/AFL, which certainly had an impact on the selection of patients for ECV, and also on the results of this analysis. Despite a methodologically clearly defined strategy of searching for arrhythmia recurrence during follow-up, there are still differences in rhythm monitoring between CIED and non-CIED patients in terms of higher sensitivity for detecting asymptomatic recurrence in patients with CIED. Although the analysis highlighted the importance of the type of atrial arrhythmia as an indication for ECV, additional analysis including the type of AFL (typical and atypical atrial flutter) was not conducted due to lack of complete data. The study analyzed therapy prescribed upon discharge from the hospital after ECV, but we do not have available data on how long this therapy was continued, nor whether there were any changes in therapy during the one-year follow-up. Although several associated factors showed relatively large odds ratios, the correspondingly wide confidence intervals indicate significant uncertainty and imprecision in the estimated effects. Therefore, these point estimates should not be overinterpreted, and the observed associations should be interpreted with caution. Finally, given that this is a retrospective, observational, single-center study, the scope of its conclusions is limited by its design.

## Conclusions

Only one in ten patients had arrhythmia recurrence within one month after successful ECV. Shorter arrhythmia duration and smaller LA size are associated with one-month sinus rhythm maintenance. In patients with implanted CIEDs, atrial arrhythmia with slow ventricular conduction requiring ventricular pacing was independently linked to short-term sinus rhythm stability. After successful elective ECV, the probability of maintaining sinus rhythm during one year, in absence of an early recurrence, is 45%. Long-term sinus rhythm maintenance in patients who were arrhythmia-free in the first month after the procedure is independently associated with smaller LA dimensions, patient's non-smoking status, CHA2DS2-VASc score less than or equal to 3, absence of prior ECV, use of antiarrhythmics both before successful ECV and at hospital discharge, and the fact that the arrhythmia episode leading to ECV was also the first arrhythmic episode overall.

## Data Availability

The raw data supporting the conclusions of this article will be made available by the authors, without undue reservation.
